# Barriers to family-centred care of hospitalised children at a hospital in Gauteng

**DOI:** 10.4102/hsag.v27i0.1786

**Published:** 2022-10-28

**Authors:** Tsholofelo C. Malepe, Yolanda Havenga, Paulina D. Mabusela

**Affiliations:** 1 Adelaide Tambo School of Nursing Science, Faculty of Science, Tshwane University of Technology, Pretoria, South Africa

**Keywords:** primary caregivers, nurses, barriers, family-centred care, hospitalisation, children

## Abstract

**Background:**

Hospitalisation is a stressful event for the admitted child and the family. The unfamiliar and stressful hospital environment could increase children’s anxiety and pain experiences. Family-centred care has the potential to promote families’ holistic health, but its implementation is limited.

**Aim:**

To describe the barriers to family-centred care at a specific hospital in Gauteng.

**Setting:**

The study was contextual and was conducted at a specific hospital situated in Gauteng.

**Methods:**

A descriptive qualitative research design was used to collect data from 11 nurses and 14 primary caregivers of hospitalised children. Purposive sampling was used. Data were collected using semi-structured interviews and analysed using qualitative content analysis. Rigour through measures to enhance trustworthiness was ensured and ethical principles related to research with human participants were adhered to.

**Results:**

Three themes indicating the barriers to family-centred care emerged from the data, namely nurse-primary caregiver relationship, primary caregiver involvement, and ward structure and policy.

**Conclusion:**

Barriers to family-centred care involved interpersonal, environmental, and managerial dimensions of the hospital environment where children received care and treatment. A need to enhance family-centred care was therefore identified in order to address relational dimensions of the nurse-primary caregiver relationship, ward structure, and revision of current policies.

**Contribution:**

The article highlights barriers to family-centred care to enable action to be taken in the clinical environment to enhance a family-centred approach and improve the hospitalisation experience for children and caregivers.

## Introduction

Hospitalisation is a stressful event for the admitted child and the family, and could be a traumatic experience if not managed effectively (Girgin & Sivri 2015; Tedford & Price 2011). Children are vulnerable as they are developmentally less able to deal with the stressful hospital environment. In addition, being ill makes it difficult for children to cope physically and emotionally (Paliadelis et al. 2005) with this unfamiliar environment and long periods of separation from their caregivers and siblings (Bonn 1994; Irlam & Bruce 2002; Leonard, Verster & Coetzee 2014). During hospitalisation, children may experience increased anxiety, pain and distress (Bonn 1994). Hospitalisation is stressful for the families as hospitalised children are cut off from their usual support systems (Tedford & Price 2011), and families experience additional stresses related to the child’s illness and their exposure to traumatic procedures (Makworo, Bwibo & Omani 2016:50). Nurses in these hospital environments play an important role in meeting the social, emotional and physical needs of the hospitalised child and the family (Coyne et al. 2011; Irlam & Bruce 2002; Zdun-Ryżewska et al. 2021). Family-centred care is a caring philosophy that could provide the framework for supporting hospitalised children and families, but its implementation remains problematic (Coyne et al. 2011).

Family-centred care is a holistic approach to planning, delivering and evaluating healthcare that is grounded in mutually beneficial partnerships among families and healthcare providers who recognise the importance of all members of the family in the life of an hospitalised child (Coyne et al. 2011; Eichner et al. 2012; Shields 2015). According to this care approach, families are fully engaged and often lead the process, while the healthcare providers act as counsellors and advisors (Irlam & Bruce 2002). When parents are involved in their children’s care, they are consulted about and included in all decisions about their children’s treatment and are more involved in clinical care provision (Kalhor et al. 2022). The extent of involvement of the parent should be negotiated during the care provided to the child (Corlett & Twycross 2006). Franck et al. (2015) described family-centred care as a healthcare delivery structure of interconnected principles and practices that recognise the significance of family members in the child’s health and well-being. Principles of family-centred care include open communication and information sharing between families and healthcare providers, shared decision-making, mutual respect, a culturally sensitive partnership and collaboration between healthcare providers and families (Abraham & Moretz 2012; Hutchfield 1999; Kuo et al. 2012; Makworo et al. 2016; Roets, Rowe-Rowe & Nel 2012). Nurses who implement family-centred care acknowledge that family is a consistent part of the child’s life, collaborate with families during the child’s care, recognise individual strengths and coping mechanism of families, share information honestly and openly and facilitate parent to parent support. Furthermore, the nurses recognise and include in nursing care the developmental needs of children and families at different stages of the lifespan, provide emotional support to families, design nursing care plans that are flexible and accessible to families. Health services, policies and programmes are designed to include families and nurses support other healthcare providers and systems to provide family-centred care (Johnson et al. 1992).

Implementation of family-centred care has benefits at various points of the care continuum. During hospitalisation, family-centred care has proven to speed up children’s recovery process (Girgin & Sivri 2015; Warren 2012). Family-centred care also makes hospital care easier by improving children’s cooperation during hospital procedures and improving their trust of healthcare providers (Kuo et al. 2012; Makworo et al. 2016; Warren 2012). When families are partners in taking care of the child, they are more empowered to care for the child after discharge, reducing the rate of readmission (Girgin & Sivri 2015; Makworo et al. 2016).

Despite the benefits of family-centred care in the paediatric context, its implementation during hospitalisation is limited (Irlam & Bruce 2002). Various studies have highlighted the challenges with the implementation of family-centred care related to the family of hospitalised children (Abraham & Moretz 2012; Butler, Copnell & Willets 2013; Kuo et al. 2012; Roets et al. 2012), the healthcare providers (Abraham & Moretz 2012; Makworo et al. 2016) and the health service or healthcare facility (Coyne et al. 2011; Lloyd, Elkins & Innes 2018; Mirlashari et al. 2019). Examples of such barriers are: unsupportive interaction between staff and caregivers, physical resources, environmental limitations (Lloyd et al. 2018) and lack of policy support for parental involvement that were restricting the nurses from implementing family-centred care (Kuo et al. 2012).

### Problem statement

At the hospital where the study was conducted, there were general ward and hospital policies about visitation and lodging. There were no policies specifically highlighting principles of family-centred care, which led the researcher to identify possible barriers to family-centred care in the specific paediatric wards as these barriers had not yet been identified. If these barriers to family-centred care in the hospital environment are identified, they could be addressed and minimised. Exploring the barriers from the perspective of the families receiving care and the nurses providing care could contribute to shaping the hospital environment to be an optimal care environment for children. Therefore, the primary researcher set out to determine the extent to which family-centred care of hospitalised children was implemented at a specific hospital in Gauteng and make recommendations for nurses and hospital management to address the identified barriers.

## Aim

The aim of the article is to describe the barriers to family-centred care at a specific hospital in Gauteng, based on an exploration of nurses’ views regarding the implementation of family-centred care and primary caregivers’ experiences during their child’s hospitalisation.

## Theoretical framework of the study

The Theory for Health Promotion in Nursing (University of Johannesburg, Department of Nursing Science 2012) was the theoretical framework of the study. The purpose of this theory is to promote the health of the individual, family, group and community (University of Johannesburg, Department of Nursing Science 2012). This theory was suitable for the study as it enabled exploration of the barriers to family centred care that emanated from the dynamic interaction between primary caregiver-child’s and the health care environments.

## Methods

### Research design

A descriptive qualitative research design was followed (Polit & Beck 2021), as this design emphasises collecting dynamic and holistic views from participants. The design enabled an exploration of nurses’ views about the extent to which family-centred care is practised in a specific hospital as well as caregiver experiences during their child’s hospitalisation.

### Setting

The study was contextual as it was conducted at a specific hospital situated in Gauteng. The hospital serves a population of approximately 120 000 people from Gauteng, North West, Limpopo, and Mpumalanga provinces (Statistics South Africa 2011). The healthcare users were from urban, semi-urban as well as rural areas, and represented a variety of cultures in South Africa. The specific hospital has a bed capacity of over 1000, including the two paediatric wards, a medical ward and a surgical ward, with a bed capacity of 78. There are also 16 outpatient clinics, including the paediatric outpatient clinic.

### Population and sampling

Two populations participated in this study, namely all nurses working in the paediatric wards and all caregivers of children who had previously been admitted to the paediatric wards. The total number of nursing personnel for both medical and surgical paediatric wards was 51 at the time of data collection. The number of children seen at the paediatric outpatient department is roughly 120 children per day, with a total of approximately 3360 children per month. The accessible population of nurses were all categories of nurses working in paediatric medical and surgical wards at the specific hospital. The accessible population (Polit & Beck 2021) of caregivers were those who visited the paediatric outpatient department after discharge for a follow-up visit with the previously hospitalised child.

Purposive sampling (Polit & Beck 2021) was used for nurses and caregivers based on specified sampling criteria. The inclusion and exclusion criteria are listed in Table 1.

**TABLE 1:** Sampling inclusion and exclusion criteria for nurses and primary caregivers.

Sampling criteria	Nurses	Primary caregivers
Inclusion criteria	Nurses from all categories, that is, professional nurses, enrolled nurses and enrolled auxiliary nurses who: had been working in a paediatric ward for a minimum of 6 months as they had enough experience in the environment were registered with the South African Nursing Council	A primary caregiver was viewed as a biological parent, foster parent, adoptive parent, aunt, uncle, grandparent or older sibling who: was aged 18 years or older was the primary caregiver of a child aged 0–12 years who had been admitted in the specific hospital in the past 6 months
Exclusion criteria	Student nurses, nursing lecturers and nurses who were on leave at the time of data collection	Primary caregivers who were stressed because of their child’s condition, for example, those whose children were critically ill, and those who were not willing to participate in the study. Primary caregivers of a premature baby were also excluded.

The views of all categories of nurses were included as family-centred care is a multi-professional approach and all nurses working in the paediatric hospital environment could participate in the identification of the barriers.

Sampling took place separately until saturation was achieved for the nurses and the caregivers, as evidenced by the repetition of themes in both groups of participants (Polit & Beck 2021).

### Data collection

Data were collected between November 2018 and September 2019 using semi-structured individual interviews (Galletta 2013) based on an interview schedule. The gatekeeper was the chief executive officer (CEO) of the hospital who provided permission for the researcher to approach the accessible population of nurses and primary caregivers. The researcher gained access to the primary caregivers through the paediatric outpatient unit manager and to the nurses through the unit managers. The questions were: ‘Tell me about your experience of having a child in hospital’ (primary caregivers) and ‘Tell me about your experiences, if any, about including the caregivers of hospitalised children in their care’ (nurses).

Interviews were conducted by the primary researcher in English and Setswana in a private room at the hospital. Interviews varied in length from 20 to 50 min depending on the amount of information the participant had to share. Interviews were audio recorded with the participants’ consent and reflective and descriptive field notes were kept. The interview schedule was piloted with six nurses and four primary caregivers prior to the actual data collection. The number of primary caregivers and nurses included in the pilot interviews were based on the participants available and willing to participate on the specific day the pilot interview was conducted. This was performed to test and improve the interview schedule and ensure that the interviewer adequately conducted the interviews. The piloted interviews were included in the data as no changes were required and as they provided dense information about their experiences regarding family-centred care. No follow-up interviews were required.

### Data analysis

Interviews were transcribed verbatim. The primary researcher conducted all interviews. Some interviews were carried out in English and others in Setswana depending on the interviewees’ preference. Interviews conducted in Setswana were translated by the primary researcher into English and checked by another first-language Setswana speaker. Field notes were added next to the appropriate sections of the transcripts. Data were analysed by means of qualitative content analysis, which is a systematic procedure to examine the content of recorded information that is relevant to the topic (Polit & Beck 2021). During this type of analysis, data are grouped into appropriate, meaningful categories (Polit & Beck 2021). Data were analysed by an independent coder and the primary researcher. The primary researcher and the independent coder met to compare and merge their independent analyses. The primary researcher and independent coder discussed any discrepancies and decided on the final categorisation of the data. Data for nurses and caregivers were analysed and described separately. Matching categories were identified. Barriers to family-centred care came to the fore in both sets of data.

### Ethical considerations

The Belmont Report’s (The National Commission for the Protection of Human Subjects of Biomedical and Behavioural Research 1979) ethical principles and guidelines for the protection of human subjects of research were used to guide the study. These principles include: respect for persons, beneficence, non-maleficence and justice (Polit & Beck 2021). Ethical clearance was obtained from Tshwane University of Technology, Research Ethics Committee, reference number: FCRE 2016/11/001 (SCI) (3) and the Gauteng Department of Health. Gatekeeper permission for the study was granted by the CEO of the specific hospital. Once all the permissions had been obtained, the primary researcher approached the unit managers of the paediatric wards and made an appointment to conduct interviews. Unit managers introduced the researcher to the potential participants but were not involved in the recruitment and selection of the participants. After the primary researcher was introduced to the primary caregivers and the nurses by the nursing manager, the primary researcher proceeded with the recruitment process in the absence of the nursing manager by introducing the study and informing potential participants whose participation was voluntary. Participants were further informed about the purpose of the study, the voluntary nature of participation and their right to withdraw from the study at any time. Participants were presented with a written information leaflet and consent letter. Consent to an audio recording or audio recording of the interviews was also sought. All interviews were anonymised with codes and were conducted in a private room in the ward or outpatient department to ensure privacy. A referral to the psychologist or counselling centre was available for those participants who might become emotional during interviews; however, this service was not needed.

## Trustworthiness

The quality of the study was enhanced by implementing specific strategies for trustworthiness based on Lincoln and Guba’s criteria (1985 in Polit & Beck 2021). To enhance the *credibility* of the study, purposive sampling was used, data saturation was ensured and participant triangulation was achieved by using two population groups, namely nurses and primary caregivers (Polit & Beck 2021). Member checking was performed at the end of each interview when the researcher summarised the content.

*Dependability* and *confirmability* of data were enhanced through well-kept documentation and transparency in the methodology, data analysis, and conclusions. *Transferability* was enhanced by obtaining responses from 11 nurses and 14 primary caregivers and by confirming that saturation had been achieved. *Authenticity* of the study was enhanced by audio recording the interviews and providing verbatim quotes when describing the findings of the study (Polit & Beck 2021).

## Findings

The sample and the findings from the interviews are described next.

### Sample

A total of 11 nurses participated in the interviews, of whom 4 were registered nurses, 4 were enrolled nurses, and 3 were enrolled nursing assistants. In all, 14 primary caregivers participated in the study. Half (*n* = 7) of the primary caregivers were in the age range of 20–29 years and the other half were 30 years and older. Half (*n* = 7) of the primary caregivers’ hospitalised children were younger than 1 year of age. The other half were between 1 and 5 years of age. Most of the children had been admitted because of medical conditions (*n* = 11). The other six children were admitted for surgical procedures. For most of the participants (*n* = 10), it was their child’s first admission. The average length of the children’s hospital stay was 16.5 days. Of the 14 primary caregivers, 6 stayed with the children and 8 commuted between home and the hospital.

### Description of findings

**FIGURE 1:**
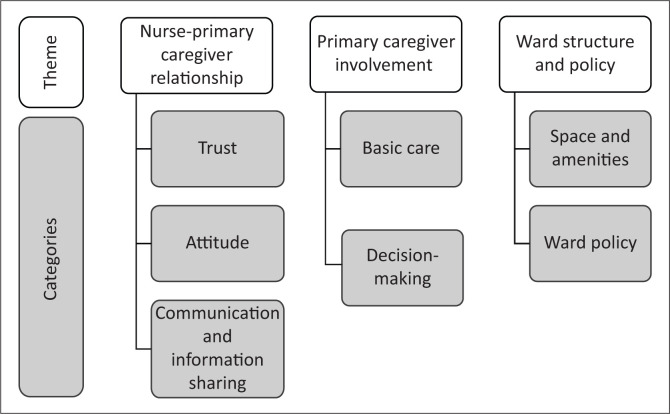
Themes and categories of the barriers to family-centred care.

Barriers to the implementation of family-centred care were identified in both sets of data, namely the nurses’ data and the primary caregivers’ data and the two sets of data were merged. The barriers to family-centred care were reflected in three themes, namely nurse-caregiver relationship, primary caregiver involvement, and ward structure and policy. Figure 1 provides a summary of the themes and categories identified. The participants’ quotes are provided unedited.

#### Nurse-primary caregiver relationship

Dynamics in the nurse-primary caregiver relationships were identified as a barrier to family-centred care. These dynamics were related to trust and interpersonal communication in the nurse–primary caregiver relationships. Nurses were of the view that they were mistrusted by the primary caregivers and perceived primary caregivers to interfere with their nursing tasks when they were present in the ward. Primary caregivers perceived negative attitudes and limited communication and information sharing by nurses during their child’s hospital stay as negative experiences, which influenced their ability to participate in their child’s care during hospitalisation.

**Trust:** Mistrust between nurses and primary-caregivers affected the quality of their relationship. Primary caregivers were uncomfortable leaving their children in the unsupervised care of nurses. One primary caregiver explained:

‘Having a child in hospital is not nice because you have to leave your family every day to visit the child in hospital and also when you leave your child in hospital with the nurses you are not sure how they will treat your child.’ (Primary caregiver 2, 25 years old, child 6 years old)

Another primary caregiver expressed concern about the treatment their child would receive when left alone in the care of nurses by saying:

‘You also worry how the nurses will treat your child. Will she just be left there to cry? Who will pick her up if she cries?’ (Primary caregiver 3, 20 years old, child 9 months old)

Similar to the primary caregivers, nurses were of the perception that they were not trusted by primary caregivers to provide competent and compassionate care to their children. One nurse explained:

‘The parents who do not stay in hospital feel that their children are being neglected, so they start lacking trust to some of the staff members and feel that their children are neglected.’ (Nurse 6, enrolled nurse, female)

In addition, nurses were of the view that their competence was questioned and they were therefore not trusted by primary caregivers as explained by the following nurse:

‘The mother will ask you something and you find that she has some knowledge about that thing, then the other person comes, then the mother asks the other person because maybe she is just testing your knowledge and does not trust you.’ (Nurse 1, professional nurse, female)

Nurses mistrusted the intentions of primary caregivers who spent extended periods of time in the wards with their children. Nurses perceived these primary caregivers as interfering with the care of other children in the ward. They felt that primary caregivers who spent a great deal of time in the ward were misinforming other primary caregivers when they came to visit their children as the following two nurses explained:

‘There was a mother whose child was very ill, who we allowed to be around the child anytime. She told the mother of the child who does not stay in hospital that her child is not taken care of, the nurses leave her to cry and do not change her nappies.’ (Nurse 4, enrolled auxiliary nurse, female)

‘It’s very difficult to deal with mothers who spend most of the time in the ward, they become inquisitive and tell other mothers false information about nurses.’ (Nurse 11, Enrolled auxiliary nurse, female)

**Attitudes:** Both nurses and primary caregivers explained how perceived attitudes towards each other impact their interpersonal relationships. Primary caregivers opined nurses as having limited empathy, being unsupportive and displaying a negative attitude towards them:

‘They do not have the heart to feel sorry for us as mothers who have sick children, they would talk to you as they please, which is not right, because they are there to care for our children.’ (Primary caregiver 5, 29 years old, child 3 months old)

‘I don’t feel the nurses are supportive and welcoming, *Ge e le gore* [*if*] all the nurses were welcoming they would all tell us their names and explain to us as parents what is going to happen during the period that the child is in hospital.’ (Primary caregiver 1, 33 years old, child 10 months old)

Another claimed that ‘nurses have a very bad attitude towards us the parents of the sick child, they would look down on us as if we are nothing’ (Primary caregiver 11, 19 years old, child 11 months old)

One of the nurses agreed that their attitudes toward primary caregivers was unsupportive by saying that:

‘The nurses’ attitudes at times can be negative towards primary caregivers. That must change. We expect them to understand when we tell them they would be leaving their children in hospital. Knowing that myself it is difficult to leave my child with someone I do know.’ (Nurse 6, enrolled nurse, female)

Nurses also explained how they had to deal with the negative attitudes of some primary caregivers. One nurse explained:

‘The babies come with a different condition, other mothers of the babies who lodge, they don’t accept the condition, they want answers from us, so they become very rude. (Nurse 5, professional nurse, female)

**Communications and information sharing:** Nurses acknowledged that information sharing is part of family-centred care and felt that they adequately shared information through health talks. Nurses further stated that vital information regarding the child’s health and prognosis was only given by doctors. Nurses explained the information they shared:

‘We share verbally, if we have something to show them, for example, [*with a*] cardiac patient we have a structure and we show them [*the primary caregivers*] the congenital abnormalities on that structure.’ (Nurse 2, professional nurse, female)

‘The children with diarrhoea and vomiting, we also give them [*primary caregivers*] health education on cleanliness and sterilising the child’s bottles and washing of hands.’ (Nurse 8, registered nurse, female)

Primary caregivers had a different experience and stated that there was limited communication and information from nurses:

‘They [*nurses*] do not talk to us they just do their jobs and leave.’ (Primary caregiver 11, enrolled auxiliary nurse, female)

‘Nurses are evil at times, they will never ask you or tell you how your child is doing.’ (Primary caregiver 13, 31 years old, child 6 months old)

‘I was told when I came to visit, that “your son is discharged” and I was given medication, [*the nurse*] never explained on how to use it. The only thing I was told was that I should bring my child for follow-up at the clinic after a month.’ (Primary caregiver 3, 20 years old, child 9 months old)

‘The nurses did not tell me what was wrong with my child, they would give me medication to give to her, they will not explain to me what the treatment is for.’ (Primary caregiver 8, 18 years old, child 5 months old)

#### Primary caregiver involvement

Primary caregiver involvement during the child’s hospitalisation was often limited to basic care. Primary caregivers had a need for more involvement and nurses acknowledged the value of primary caregiver participation for more than basic care. In terms of involvement in decision-making, this was often limited to consent for procedures.

**Basic care:** Involvement limited to basic care by primary caregivers during their child’s hospitalisation was perceived to be a barrier to family-centred care. Both nurses and primary caregivers agreed that, when primary caregivers were present in the ward, their involvement was limited to basic care:

‘The positive thing is that they are helping us with bathing, feeding their babies. You will find that sometimes we are short-staffed in the ward and we cannot take care of all the children.’ (Nurse 6, enrolled nurse, female)

‘They do bath their children, feed them and change their napkins [*nappies*]. For those children who can walk, usually when the mother is around during visiting time, they do take them to the toilet.’ (Nurse 5, professional nurse, female)

‘I was very much involved, every time when I get to the hospital, I would bath my child, change her in clean clothes and feed her.’ (Primary caregiver 5, 29 years old, child 3 years old)

‘I was bathing, breastfeeding, feeding and changing napkin for my son.’ (Primary caregiver 7, 22 years old, child 7 months old)

Primary caregivers had a need for deeper involvement than just basic care as explained by the following participants:

‘I want to be part of the care of my child, when they give her medication, they should give it to me to give it to her.’ (Primary caregiver 11, 19 years old, child 11 months old)

‘I wish the nurses could show us everything they are doing or giving to my child, so that even at home when she starts getting sick, I know what to do.’ (Primary caregiver 12, 41 years old, child 1 year old)

‘There is no transparency, the nurses will take your child and she comes back with that thing they have put into her to give water [*intravenous infusion*], I want to be part of that.’ (Primary caregiver 14, 25 years old, 1 year and 7 months)

Nurses acknowledged the value of primary caregivers’ involvement during the child’s hospitalisation, which resulted in faster recovery and reduced readmissions as explained by two nurses:

‘My experience is that when we include the caregivers and the family we observed that the child gets much better quicker because we are all involved in the caring of the child.’ (Nurse 2, professional nurse, female)

‘The positive experience is that when the mothers are around they can learn how to feed their children, especially the malnutrition children. It will also help us to reduce readmissions.’ (Nurse 3, enrolled auxiliary nurse, female)

**Decision-making:** Both nurses and caregivers agreed that there was limited involvement of primary caregivers in decision-making during their child’s hospitalisation. Nurses agreed that health professionals were the main decision makers and that primary caregivers had limited say. Two of the nurses explained:

‘Most of the time the doctors and the multidisciplinary team are the ones who make most of the decision[*s*] especially regarding the condition of the child. The parents have a limited say.’ (Nurse 1, professional nurse, female)

‘Yes, they do [*participate in decision-making*] but not most of the time. Only if the child has to go for an operation the doctor will inform the parents about the operation, they will be given a consent to agree about the child’s operation.’ (Nurse 7, enrolled nurse, female)

Primary caregivers explained that their involvement was limited and that they were often only involved in giving consent to procedures:

‘No there is no decision that I made, the nurses and doctors were the one’s making decisions regarding the treatment of the child.’ (Primary caregiver 4, 34 years old, child 1 year and 9 months old)

‘I was told that my child is going for an operation to remove tonsils and I have to sign an agreement form, which I signed, other than that nothing was communicated.’ (Primary caregiver 9, 20 years old, child 1 year and 11 months old)

#### Ward structure and policies

The ward structure and polices were identified by both nurses and primary caregivers as a barrier to family-centred care. The spaces and amenities were limited in accommodating primary caregivers and families being present. Visitation policies were viewed as a barrier to implementing family-centred care.

**Space and amenities:** Nurses and primary caregivers agreed that because of limited space in the paediatric wards, caregivers and other family members could not be accommodated. Furthermore, because of the limited space when primary caregivers were included, privacy and confidentiality were compromised. As nurses explained:

‘If the hospital can extend the ward because we are having a challenge that there are lots of mothers who want to be with their children in hospital but we do not have enough space and beds.’ (Nurse 6, enrolled nurse, female)

‘The ward structure should be changed in a way that the families can be with their children in hospital.’ (Nurse 10, enrolled nurse, female)

Primary caregivers had similar experiences and stated that the ward structure did not meet the needs of the hospitalised child and their families. Participants explained that the ward was not large enough to accommodate the admitted child and their family because of its structure:

‘The ward environment is very small to be child and family friendly. When other family members visit you have to take chairs from other children’s beds and the space is not enough.’ (Primary caregiver 7, 22 years old, child 7 months)

‘It is not child and family friendly, my experience with the ward is that even if you want to play with your child outside the bed, the space is not enough so the child is confined to the bed most of the time.’ (Primary caregiver 8, 18 years old, child 5 months old)

The limited space led to a lack of privacy in the ward. The ward was open plan and there was no privacy between the children’s beds, as the beds were right next to each other:

‘The ward restricts your movement, there is nothing to do, the cubicles do not have a TV, if you play music to your child, you feel like you are disturbing others. It is an open plan ward, with no privacy.’ (Primary caregiver 9, 20 years old, child 1 year and 11 months old)

‘There are no curtains in between the bed.’ (Primary caregiver 5, 29 years old, child 3 years old)

‘The mothers are very inquisitive about other patient’s information, there is no privacy in the ward, and they would be listening to other patients’ information.’ (Nurse 3, auxiliary nurse, female)

In addition to the limited space, the ward structure was not child friendly. For example, the bathrooms were not suitable for small children and the ward was viewed by primary caregivers as not being homely:

‘The ward should be restructured that it accommodates the siblings and fathers to make it feel homely.’ (Primary caregiver 13, 31 years old, child 6 months old)

‘The beds are not suitable for the child and the facility in the bathroom does not accommodate children.’ (Primary caregiver 6, 24 years old, child 2 years old)

#### Ward policy

Some of the ward policies related to parental involvement and visitation seemed to be a barrier to practising family-centred care. The visiting hours were viewed as rigid, and only certain primary caregivers, such as breastfeeding mothers, were allowed to stay at the hospital. In addition, age restrictions limited visitation by siblings.

The rigid visiting times were viewed as a barrier to family-centred care by both nurses and primary caregivers as explained by the following participants:

‘The hospital policy is restricting those mothers who are not breastfeeding and their children are older than 6 months. They can only visit the ward at 15:00 during visiting time.’ (Nurse 10, enrolled nurse, female)

‘Even the mothers who are staying in hospital do not spend the entire time with their children, and they want to be next to their children all the time.’ (Nurse 11, enrolled auxiliary nurse, female)

‘The visiting time should not only be at 3, we should be allowed to visit anytime.’ (Primary Caregiver 2, 25 years old, child 6 years old)

‘I would have loved to be there for my child every moment, but because of restrictions, no. One day I just went there during strange times and I was not allowed to see my child.’ (Primary caregiver 5, 29 years, child 3 years old)

Furthermore, only breastfeeding mothers whose children were 6 months old or younger were allowed to stay at the hospital when their child was in hospital. The policy did accommodate mothers with children who were terminally ill and needed more flexibility:

‘Maybe the policy should be changed to accommodate every mother whose child is admitted in hospital to visit anytime irrespective of the child’s age.’ (Nurse 11,enrolled auxiliary nurse, female)

‘Maybe the lodging policy can be reviewed to accommodate all mothers, for example, the 7-month-old baby who is breastfeeding and the mother just introduced him to solids and the mother must travel to hospital every day to be there for 3-hourly feeds and also the nurse’s attitude must change.’ (Nurse 5, professional nurse, female)

Finally, the age restrictions for visiting siblings made it impossible for the hospitalised children to spend time with their siblings:

‘We only allow siblings who are older than 12 years to avoid them from getting infection from the sick children.’ (Nurse 9, enrolled nurse, female)

‘There should be a waiting room to accommodate the siblings to the sick child, the mother can be able to take the sick child to the waiting room so that the other siblings can play with her, to avoid that separation from his or her siblings.’ (Nurse 11, enrolled auxiliary nurse, female)

‘Yes I would have loved for her siblings to visit her while in hospital, but they were not allowed, maybe they should make a room for the siblings to visit the child in hospital.’ (Primary caregiver 8, 18 years old, child 5 months old)

‘The other siblings will be asking you about his whereabouts and they want to visit me and the child in hospital, which cannot be done, they are underage and will not be allowed to the ward.’ (Primary caregiver 10, 34 years old, child 3 years old)

The three themes, namely nurse-caregiver relationship, primary caregiver involvement, ward structure and policy were described and supported by quotes from both the primary caregivers and nurses.

## Discussion

The findings of the interviews with nurses and primary caregivers revealed similar barriers to family-centred care, namely dynamics in the nurse–primary caregiver relationship, involvement of the primary caregiver, structure of the ward and ward policy. These barriers to family-centred care concurred with the findings by Lloyd et al. (2018), who found that unsupportive interaction between staff and mothers, physical resources and environmental limitations restricted nurses from implementing family-centred care. Mirlashari et al. (2019) found that organisational limitations were a challenge in implementing family-centred care. There was no in-service education about family-centred care and insufficient facilities and organisational infrastructure did not support the implementation of family-centred care. There were therefore challenges to the implementation of family-centred care in paediatric wards.

The dynamics in the nurse-primary caregiver relationship that were barriers to family-centred care were perceived mistrust of nurses by primary caregivers, perceived interference by primary caregivers, nurses’ attitudes and limitations in communication and information sharing by nurses. To establish a trusting relationship in the nurse-primary caregiver relationship, an approachable attitude, open communication, and clear information sharing are essential (Coyne et al. 2011; Zegwaard et al. 2017). Trust between nurses and primary caregivers is essential to enable primary caregivers to participate in their child’s care. Richards et al. (2017) reinforced that listening to parents is important in allowing them to work hand-in-hand with nurses and create a trusting relationship. Coyne et al. (2011) also found that nurses felt that building a good relationship and trust with primary caregivers by listening to their concerns was important to enhance family-centred care.

Primary caregivers expressed that they experienced negative attitudes and limited communication and information sharing from nurses. In the study by Coyne et al. (2011), conducted in children’s units in Ireland, nurses suggested that clear, understandable, unbiased, timely information was essential for promoting family-centred care. These authors also endorsed that communication plays a significant role in terms of keeping parents updated at all times about every aspect of the child’s care and empowering parents to be as involved as possible (Coyne et al. 2011). The study conducted by Richards et al. (2017) showed similar findings, suggesting that parents wanted the nurses to listen to them, answer their questions, address their problems, and include their knowledge in the treatment plan. In addition, Richards et al. (2017) stated that parents want clear, truthful and complete information to effectively participate in decision-making and physically caring for their children. Adequate information enables families to make informed decisions about their child’s care and therefore impact their ability to be involved in the child’s care and treatment (Coyne et al. 2011).

According to Shields (2015), family-centred care will work only if the health professional can adapt all communication methods to accommodate the family in caring for the hospitalised child. Stern et al. (1991 cited by Butler et al. 2013) found that nurses were not a source of information to the primary caregivers and often felt that no one had taken any responsibility to communicate with the family. Similarly, in this study, the information the nurses shared with the primary caregivers was very limited and generally in the form of health education. In addition, some primary caregivers indicated that no information was given to them.

Involvement in care and decision-making is an important component in promoting family-centred care in paediatric wards. According to Butler et al. (2013), parental involvement is a predominant aspect in caring for the sick child in hospital. Shields (2015) claimed that the healthcare organisation’s most significant role is to provide opportunities for families to support the hospitalised child and families’ involvement in the implementation of family-centred care in hospitals should be promoted. According to the Children’s Act (South African Government 2005), a primary caregiver acts on behalf of the child, as the child is regarded as a minor. Therefore, the primary caregiver must give consent for treatment and be informed about the child’s condition and prognosis to make an appropriate decision on behalf of the child. The Children’s Act (South African Government 2005) further stipulates that primary caregivers have the right to advocate on behalf of the child by making informed decisions in relation to the child’s health. In a study by Mirlashari et al. (2019) about the challenges of implementing family-centred care in neonatal care units, nurses stated that doctors had power over both the nurses and families in terms of decision-making. Because the doctors’ decisions were final, nurses avoided involving the families in making decisions for the hospitalised child as they would not be heard.

The study by Shields (2015), about nurses’ and families’ understanding of family-centred care, their perceptions and whether family-centred care makes a difference in caring for the hospitalised child, supported the findings of the present study, as the author found that primary caregivers were mostly involved in basic care related to their child. Shields (2015) explained that nurses believed that primary caregivers should stay in the hospital to provide basic care to their hospitalised child.

The structural environment impacts family-centred care. In this study, the structural environment was also a barrier for the nurses and primary caregivers because of inadequate ward space and lack of privacy. In a study by Lloyd et al. (2018), on the barriers and enablers of patients and family-centred care in an Australian acute care hospital, it was suggested that physical resources and environmental factors restrained the implementation of family-centred care. Some of these resources and environmental factors included not having enough space between the children’s beds and not having comfortable chairs available for parents. As was the case in this study, Shirazi et al. (2015) found that the lack of privacy restricted private conversations between the nurses, children and family members. Furthermore, similar to this study, Shields (2015) determined that the ward was small and not designed for family-centred care. It therefore became difficult for the nurses to accommodate families.

Policies can act as enablers or barriers to family-centred care. In this study, the policies regarding visiting times and staying in hospital with children were seen as rigid. These findings regarding policy restrictions concurred with the studies by Mirlashari et al. (2019) and Kuo et al. (2012). Mirlashari et al. (2019) conducted a study to investigate physicians’ and nurses’ perspectives on the challenges of implementing family-centred care in a neonatal intensive care unit, while Kuo et al. ’s (2012) study was about a lack of organisational arrangement and lack of policy support for parental involvement in caring for the hospitalised child. Both studies revealed challenges in implementing family-centred care related to the non-existence of family-centred ideas of care in the hospital’s strategic plan.

### Limitations

The primary researcher focused on primary caregivers and nurses and excluded other healthcare providers from the study. Further research should include other members of the multidisciplinary team who all participate in the holistic care provision of children and families during hospitalisation. Most of the primary caregivers who participated in the study were biological mothers of the hospitalised children. More experiences of primary caregivers who were not biological mothers and other members of the family could be included. Their views will be equally important in the application of a family-centred care philosophy and in making recommendations that include all family members.

### Recommendations

In this study, the nurses and primary caregivers indicated that there were obstacles to the implementation of family-centred care, which made it difficult for both nurses and primary caregivers to collaborate, thus hindering the implementation of family-centred care of the hospitalised child in paediatric wards. Based on the findings, it is recommended that the hospital revise paediatric ward policies and ward guidelines based on current evidence-based practices regarding holistic care and the benefits of family-centred care. The voice of the community should be included in these revisions. Continuous in-service training programmes should be a requirement for all nurses to enhance their competence in practising family-centred care, promoting effective and empathic communication and information sharing and building trusting relationships with primary caregivers and families. Hospitals should evaluate the structural environment and obtain feedback from the healthcare users of the facility on how to make the environment more family friendly. Primary caregivers should also be educated about family-centred care and their rights as healthcare users. Furthermore, family-centred care should be addressed in the nursing curriculum to promote and assist in facilitating the implementation of family-centred care by nurses of all categories.

## Conclusion

This study presented barriers to family-centred care of hospitalised children at a specific hospital in Gauteng from the point of view of the nurses who provide care and the primary caregivers of children who use the healthcare services. Interpersonal and environmental barriers were identified that negatively affected the practice of family-centred care in the paediatric wards of the specific hospital. The nurse-caregiver relationship is central to enhancing family-centred care because, through effective communication and information sharing, primary caregivers can be more enabled to participate in and make informed decisions about their child’s care. The interpersonal, structural and managerial environment should be mobilised towards creating more inclusive environments for families that promote holistic care and treatment of children in the hospital environment.
